# In Memoriam

**Published:** 1880-02

**Authors:** J. H. Redding, F. B. McRae, R. J. Bruce, K. Davis, J. P. McCurry, W. F. Westmoreland, R. O. Cotter

**Affiliations:** Committee; Committee; Committee; Committee; Committee; Ch'm.; Sec'y


### IN MEMORIAM.

The Atlanta Medical College has sustained another loss in the death of an estimable member of the class of students. In the last number of our JOURNAL we published the death of an esteemed member of the faculty, and it becomes our painful duty to chronicle the death of Mr. F. M. Jones, of Cherokee county, Georgia, a laborious, upright and popular member of the class of 1879–80. The following action was had by the class:

At a called meeting of the students on Saturday afternoon, the following gentlemen of the class were appointed to accompany the remains to the railroad station of the family of the deceased:

J. W. Holton, of Ohio; P. R. Bradford, of Georgia; W. R. Hollingsworth, of South Carolina; H. L. Logan, of Arkansas; J. Blaisdell, of Maine; E. B. Moore, of Texas.

On Monday morning a meeting of the entire class was held at the college, Prof. W. F. Westmoreland being invited to take the chair and R. O. Cotter appointed to act as secretary.

Dr. Westmoreland explained the object of the meeting to the class, it being to take preliminaries for paying the proper respect due to the memory of their much loved and lamented friend and fellow-student.

The Rev. Dr. D. W. Gwin, of this city, a member of the class, being unanimously requested to make some remarks touching the character of the deceased, responded in a short extempore address, replete with beautiful thoughts. Among other things he impressed the young men with the mutability of all earthly plans and honors. He dwelt well upon the fact that man proposes but God disposes. Here was a young man who, less than two weeks ago, was a very type of manly health and vigor. Thoroughly upright and conscientious, he was a general favorite in the class. He was happy at the prospect of so soon being able to go forth and begin the noble work for which he would have been so well fitted. In a few days only he would have received his diploma. But the "Great Physician" saw fit to give him his diploma from a nobler than any earthly institution, and sent him to work in a grander, happier sphere than this.

On motion, the chairman appointed the following members of the school as a committee of five to prepare suitable resolutions:

Messrs. J. H. Redding, of Texas; K. Davis, of Georgia; F. B. McRae, of Georgia; R. J. Bruce, of Georgia; S. J. McCurry, of Alabama.

The name of the chairman was also, by request of the class, added to the number.

The following was the report of the committee on resolutions:

WHEREAS, God in His infinite wisdom and wise providence has seen fit to call from our midst our beloved friend and fellow-student, Mr. F. M. Jones, of Cherokee county, Georgia.

*Resolved*, That while we deplore his loss to us, and what we deem would have been of so much benefit to humanity, we yield submissively to the will of Him who "doeth all things well."

*Resolved*, That we ever honor him in our memories for his combined qualities of morality, temperance, studious efforts, laudable attainments and gentlemanly deportment as a student of medicine.

*Resolved*, That we tender our most sincere sympathies to his beloved and bereaved wife and fatherless children, and his sorrowing mother.

*Resolved*, That each student wear a piece of crape, as a token of respect, until the close of the session.

*Resolved*, that these resolutions be published in the Atlanta *Constitution* and the ATLANTA MEDICAL AND SURGICAL JOURNAL, and a copy of each be forwarded to the family of the deceased.

[Signed] J. H. REDDING,
F. B. MCRAE,
R. J. BRUCE,
K. DAVIS,
J. P. MCCURRY,
W. F. WESTMORELAND,
*Committee.*

W. F. WESTMORELAND, Ch'm.

R. O. COTTER, Sec'y.

